# Personal Aging Experiences and Aging-Related Beliefs – Projection Processes in Daily Life and Multiple Countries.

**DOI:** 10.1093/geroni/igaf122.1846

**Published:** 2025-12-31

**Authors:** Maria Wirth, Anna Kornadt, Shevaun Neupert, Reyyan Can, Amit Shrira, Yuval Palgi, Klaus Rothermund

**Affiliations:** Friedrich Schiller University Jena, Jena, Thuringen, Germany; University of Luxembourg, Esch-sur-Alzette, Grevenmacher, Luxembourg; North Carolina State University, Raleigh, North Carolina, United States; North Carolina State University, Raleigh, North Carolina, United States; Bar-Ilan University, Ramat Gan, HaMerkaz, Israel; University of Haifa, Haifa, HaZafon, Israel; Friedrich Schiller University Jena, Jena, Thuringen, Germany

## Abstract

Views of aging, including personal aging perceptions and beliefs about characteristics and behaviors of older adults, impact behavior and developmental outcomes in later life. While most research focuses on how aging-related beliefs become internalized into personal aging experiences, aging perceptions can also affect aging-related beliefs, indicating a projection or “externalization” of personal aging experiences. Put differently, aging-related beliefs are not immune against one’s own age-related experiences. This study aimed to understand how personal aging experiences affect aging-related beliefs in a daily context and across multiple countries. Participants in Germany (N = 199, 50-91 years), Türkiye (N = 50, 50-90 years), USA (N = 71, 54-85 years), and Israel (N = 78, 50-88 years), reported on their daily experiences of age-related gains and losses, and on their endorsement of ageist attitudes for 14 consecutive days. Projecting own aging experiences should influence endorsement of aging-related beliefs. Specifically, experiencing more losses and fewer gains should result in endorsing more ageist attitudes, both daily and overall. Our results indicated partial support for the projection hypothesis. Across investigated countries, reporting above-average losses was related to higher endorsement of ageist attitudes. In contrast to the idea of projection, on days with more losses, endorsement of ageist attitudes was lower. Gains were unrelated to endorsement of ageist attitudes in all countries. Our results highlight the impact of negative own aging experiences for shaping beliefs about (other) older adults. Interventions targeting aging-related beliefs, thus, need to consider personal aging experiences.

